# *Catechol-O-Methyltransferase (COMT) Val*^108/158 ^*Met *polymorphism does not modulate executive function in children with ADHD

**DOI:** 10.1186/1471-2350-5-30

**Published:** 2004-12-21

**Authors:** Evan Taerk, Natalie Grizenko, Leila Ben Amor, Philippe Lageix, Valentin Mbekou, Rosherie Deguzman, Adam Torkaman-Zehi, Marina Ter Stepanian, Chantal Baron, Ridha Joober

**Affiliations:** 1 Department of Psychiatry, McGill University and Douglas Hospital Research Centre, Montreal, Quebec, H4H 1R3, Canada; 2 Department Neurology and Neurosurgery, McGill University and Douglas Hospital Research Centre, Montreal, Quebec, H4H 1R3, Canada; 3 Department of Human Genetics, McGill University and Douglas Hospital Research Centre, Montreal, Quebec, H4H 1R3, Canada

## Abstract

**Background:**

An association has been observed between the *catechol-O-methyltransferase *(*COMT*) gene, the predominant means of catecholamine catabolism within the prefrontal cortex (PFC), and neuropsychological task performance in healthy and schizophrenic adults. Since several of the cognitive functions typically deficient in children with Attention Deficit Hyperactivity Disorder (ADHD) are mediated by prefrontal dopamine (DA) mechanisms, we investigated the relationship between a functional polymorphism of the *COMT *gene and neuropsychological task performance in these children.

**Methods:**

The *Val*^108/158 ^*Met *polymorphism of the *COMT *gene was genotyped in 118 children with ADHD (DSM-IV). The Wisconsin Card Sorting Test (WCST), Tower of London (TOL), and Self-Ordered Pointing Task (SOPT) were employed to evaluate executive functions. Neuropsychological task performance was compared across genotype groups using analysis of variance.

**Results:**

ADHD children with the *Val/Val*, *Val/Met *and *Met/Met *genotypes were similar with regard to demographic and clinical characteristics. No genotype effects were observed for WCST standardized perseverative error scores [F_2,97 _= 0.67; p > 0.05], TOL standardized scores [F_2,99 _= 0.97; p > 0.05], and SOPT error scores [F_2,108 _= 0.62; p > 0.05].

**Conclusions:**

Contrary to the observed association between WCST performance and the *Val*^108/158 ^*Met *polymorphism of the *COMT *gene in both healthy and schizophrenic adults, this polymorphism does not appear to modulate executive functions in children with ADHD.

## Background

Attention Deficit Hyperactivity Disorder (ADHD) is a childhood psychiatric disorder characterized by symptoms of inattention, impulsivity and motor hyperactivity afflicting 6–8% of school-aged children in North America [1,2]. Although ADHD is a disorder with complex and heterogeneous etiology, genetic factors appear to play a significant role in predisposing and perpetuating the development of the disorder as evidenced by twin [3,4], family [5-7], and adoption studies [8]. Association studies have implicated several susceptibility loci including a 40-base pair (bp) allele of the Variable Number of Tandem Repeats (VNTR) polymorphism of the *SLC6A3 *gene [9] and a 48-bp repeat polymorphism of the *DRD4 *gene [10]. Attempts to replicate these findings have met with modest success possibly owing to the clinical heterogeneity characteristic of the disorder [11]. One method that may act to augment the strength of these associations would be to identify endophenotypic intermediates conferring risk for the development of ADHD rather than attempting to identify direct linkages between genetic variations and the behavioural manifestation of the disorder.

Theories of dysregulated dopamine (DA) pathways in ADHD have been supported by the efficacy of dopamine agonists in reducing the core symptoms of the disorder [12]. The mesocortical DA pathway appears to be integral to prefrontal cortex (PFC)-mediated cognitive functioning, specifically working memory [13], through the enhancement of task-related neural activity via D1 receptor activation [14]. Both PET [15] and SPECT [16] imaging studies support a neuromodulatory role for DA in the PFC during tasks of executive function. In addition, administration of DA agonists to the rat PFC acts to enhance working memory in these animals [17]. Consistent with this line of thinking, children with ADHD show deficits in performance of tasks of executive function [summarized in a meta-analysis by Sergeant et al. (2002)] [18] and significant improvement of performance under methylphenidate [19,20]. These findings have prompted the hypothesis that the overt symptoms of ADHD are the manifestation of an underlying deficiency in a range of PFC-mediated cognitive domains, including working memory, planning, and set shifting, collectively regarded as executive function [21-23].

The hypothesized role of a dysfunctional mesocortical dopaminergic pathway in the development of symptoms of ADHD has encouraged the investigation of candidate genes involved in this pathway including *SLC6A3 *[9], *DRD4 *[10] and, more recently, the *catechol-O-methyltransferase *(*COMT*) gene [24]. The *COMT*, encoded by a gene located on chromosome 22q11, catalyzes the degradation of catecholamines, most importantly DA [25]. A functional polymorphism of this gene, involving a substitution of Valine (*Val*) for Methionine (*Met*) at codon 108/158 (*Val*^108/158 ^*Met*), results in a 4-fold variation in enzyme activity, with individuals homozygous for either the *Val *or *Met *allele exhibiting either reduced or preserved levels of DA respectively [26]. Although the dopamine transporter (DAT) is the predominant means of DA termination in most dopaminergic neurons [27], considerable evidence exists to suggest that the DAT may play a reduced role within the PFC [28-32], where other clearance mechanisms may be implicated. Comparison of DA metabolite levels within discrete brain loci in both rats [33] and monkeys [34], as well as the measurement of DA levels in *COMT *knock-out mice [35], suggest an important functional role for *COMT *in the PFC. If *COMT *is indeed inextricably linked to DA metabolism within the PFC, it is reasonable to assume that variations in enzyme activity, as dictated by the *Val*^108/158 ^*Met *polymorphism, may modulate the performance of tasks of executive functioning in healthy individuals, as well as individuals with reduced PFC basal dopamine levels. In support of this assumption, associations have been reported between the *Val*^108/158 ^*Met *polymorphism and performance on the Wisconsin Card Sorting Test (WCST) in healthy adults [36,37]. In adults with Schizophrenia, a disorder characterized by dopaminergic hypofrontality [38], associations have also been observed between the *COMT *polymorphism and WCST performance [39-41]. Although one study reported an association between the *COMT *polymorphism and ADHD using a haplotype relative risk design [24], this study failed to investigate any indices of executive function and several other studies failed to replicate this finding [3,42-44].

Given the putative role of *COMT *in DA metabolism within the PFC [33-35], we hypothesized that the *Val*^108/158 ^*Met *polymorphism of the *COMT *gene will be associated with alterations in performance on tasks of executive function, a behavioural index of PFC integrity and function [45]. Since dysfunctional DA neurotransmission [46] and deficient neuropsychological task performance [18] are both characteristic of children with ADHD, we further hypothesized that this association would be evident within this particular clinical population. Specifically, ADHD children expressing the high enzymatic activity *Val *allele (H), resulting in reduced PFC DA neurotransmission [26], will show more pronounced deficits in neuropsychological task performance than their low enzymatic activity *Met *allele (L) counterparts. In order to test this hypothesis, we used three measures of executive function: the WCST [47], a measure of set-shifting ability capable of differentiating between ADHD children and controls [18] and associated with the COMT polymorphism in normal [36,37] and schizophrenic adults [39-41]; the Tower of London (TOL) [48], a measure of planning ability, which consistently differentiates ADHD children from controls [18], and the Self-Ordered Pointing Task (SOPT) [49], a measure of working memory also capable of differentiating between ADHD children and controls [18].

## Methods

### Subjects

118 children were recruited from the Disruptive Behaviour Disorders Program and the children outpatient clinic at the Douglas Hospital. They were referred to these specialized care facilities by school principals, community social workers, and paediatricians.

Inclusion criteria required children to be between the ages of 6 and 12 years of age, meeting DSM-IV diagnosis criteria for ADHD [50]. Diagnosis of ADHD was based on a structured clinical interview of parents using the DISC-IV (parental report) [51], school reports, teacher interviews, and clinical observation of the child. In the majority of cases, mothers were the primary informants for the collection of clinical information. Written reports from the child's school were also available in the majority of cases. Parents completed the Child Behavioural Checklist (CBCL) [52], a scale that assesses a variety of behavioural domains, and the Conners' Global Index for parents (CGI-P) [53]. Teachers also completed the Conners' Global Index (CGI-T) [54]. Assessments were made while children were free of medication. Exclusion criteria included a history of mental retardation, with an IQ less than or equal to 70 as measured by the WISC-III [55], and history of Tourette Syndrome, pervasive developmental disorder, psychosis or any medical condition or impairment that may interfere with the child's ability to complete the study.

### Neurocognitive assessment

A comprehensive neuropsychological test battery assessing different aspects of the central executive functions was administered to all children by trained research personnel. All children were assessed subsequent to a one-week medication "wash-out" period. Children were permitted to take breaks upon request and, in some cases, testing was carried out over two sessions. On average, the testing procedure lasted 1.5 hours. The research protocol was approved by the Research Ethics Board of the Douglas Hospital. Parents were explained the study and provided written consent. Children were also explained the study and gave their assent to participate as well.

Tests were selected according to their ability to tap into various performance domains of executive function. We restricted the number of tests in each domain in order to balance comprehensiveness with the co-operation of patients. Abstraction and concept formation were evaluated by means of the WCST (perseverative errors) [47]. In this task, children are required to sort cards according to three different criteria (colour, number, or shape of signs presented on cards). Feedback on whether the child achieved a correct or incorrect match is given after each trial. The matching criterion changes after ten consecutive correct matches and the child has to identify the new matching criterion using the feedback (correct/incorrect) provided to them. Evidence of the reliability and validity of the WCST with various normal and clinical populations has been reported in several studies [18]. Planning capacity was evaluated using the TOL [48]. This test is used to assess planning and problem solving aspects of executive functioning. The validity and reliability of the TOL has been reported in numerous studies [18]. Standardized administration and scoring procedures as well as normative data have been developed for paediatric populations [56]. Visual Working Memory was evaluated using the abstract version of the SOPT [49]. In this task, series of matrices of 6, 8, 10, and 12 images are presented to the child. The child is asked to select, by pointing, one different image on each page. Errors occur when the child points to images previously selected on the preceding pages. Each set is presented to the child three times. Successful performance on this task involves working memory as well as planning and monitoring skills. Shue & Douglas (1992) have reported significant differences in performance between ADHD children and normal controls on the SOPT [57].

### Molecular genetics

The *Val*^108/158 ^*Met *polymorphism of the *COMT *gene was genotyped using a PCR based method as previously described [26]. The PCR was performed in a 25 μl total reaction volume containing 1X PCR buffer, 200 uM dNTPs, 200 ng of primers (5'-GCGATGGTGGCACTCCAAGC; 5'-TTGGAGAGGCTGAGGCTGAC), 1 unit of Taq DNA polymerase, and 100 ng of genomic DNA. PCR products were electrophoresed on agarose-TAE gel along with 1 kb ad 100 bp DNA ladders, visualized under UV-light and coded according to the length of the PCR product. Genotypes were called by two independent and experienced technicians who were blind to all clinical data. No disconcordance in any of the readings was noted. Children were stratified according to genotype only after all neuropsychological task data was collected.

### Statistical analyses

The *Val*^108/158 ^*Met *polymorphism consists of both the low-activity *Met *(L) and high-activity *Val *(H) alleles. Subjects were stratified into three groups: two homozygous genotype groups (LL, HH) and one heterozygous genotype group (HL).

A one-way analysis of variance (ANOVA) where genotype (LL, HL, HH) was the independent variable and neuropsychological task performance (standardized WCST perseverative error score, standardized TOL total item score) was the dependent variable was performed. For the SOPT, no normalized scores are available and testing procedures involve several levels of difficulty (4). We therefore used a two-way, repeated measure, mixed design analysis of covariance (ANCOVA), where genotype and level of task difficulty were the between and within subjects independent variables, respectively, neuropsychological task performance (SOPT raw error score) was the dependent variable, and age was the covariate. As the TOL also involves multiple levels of task difficulty (12), we repeated the analysis for this test using the same statistical approach as that applied to the SOPT. A one-way ANCOVA, where genotype was the independent variable and age was the covariate, was performed on all other non-standardized measures of neuropsychological task performance (WCST number of categories completed, WCST number of trials to first category, TOL number of problems solved).

An investigation of linkage and within-family association between quantitative phenotypes (standardized WCST perseverative error score, standardized TOL error score, and SOPT error score) was conducted utilizing the Quantitative Trait Disequilibrium Test (QTDT) statistical software package [58].

## Results

Table 1 shows clinical and demographic information for the children stratified according to genotype [n = 23 for LL (19.5%), n = 66 for HL (56.0%) and n = 29 for HH (24.5%)]. The three groups were similar with regard to age, average household income, severity of behavioural problems as assessed by the CBCL, and mean number of inattention items, mean number of hyperactivity items and distribution of ADHD subtypes according to the DISC-IV. No significant differences existed between the groups in IQ as measured by the WISC-III. Our sample was characterized by a high prevalence of comorbid disorders, particularly oppositional defiant disorder and conduct disorder. The frequency of these disorders was equally distributed between the genotype groups. The proportion of subjects who had never received medication for ADHD within each genotype group was also remarkably similar. Although a significant effect of gender was observed between genotype groups (χ^2 ^= 7.39; df = 2, p = 0.02), this result was treated as a type I error (false positive) due to the absence of female subjects with the HH genotype and given the relative lack of female representation across all genotype groups. However, given the previously observed association between gender and several polymorphisms at the *COMT *loci [59], increasing the sample size to achieve a more comparable gender representation and distribution would be a valuable revision to the present design.

**Table 1:** Demographic and clinical characteristics of children with ADHD separated according to COMT genotype

	LL (23)	HL (66)	HH (29)	p-value
Gender (M/F)	20/3	52/14	29/0	χ^2 ^= 7.39, df = 2 p = 0.02
Age	9.2 (2.0)	9.0 (1.8)	9.3 (1.7)	F_2,115 _= 0.21, p = 0.81
IQ	97.2 (13.7)	97.5 (13.5)	95.6 (13.8)	F_2,98 _= 0.17, p = 0.84
CBCL (total score)	68.0 (9.8)	70.9 (10.4)	68.9 (8.9)	F_2,112 _= 0.87, p = 0.42
Income (% less than 20 K)	32 %	42 %	48 %	χ^2 ^= 1.39, df = 2 p = 0.50
DISC-IV Inattention Items	7.3 (1.5)	6.9 (2.2)	7.2 (2.3)	F_2,113 _= 0.46, p = 0.63
DISC-IV Hyperactivity Items	5.9 (2.4)	6.4 (2.3)	6.4 (2.7)	F_2,113 _= 0.33, p = 0.72
DISC-IV ADHD Subtype (I/H/C)	10/3/10	14/13/39	7/3/19	χ^2 ^= 5.68, df = 2 p = 0.22
Comorbid ODD	13/23	50/66	20/27	χ^2 ^= 3.21, df = 2 p = 0.20
Comorbid CD	5/23	27/64	8/27	χ^2 ^= 3.57, df = 2 p = 0.17
Never Medicated	11/22	38/62	18/28	χ^2 ^= 1.17, df = 2 p = 0.56

CBCL = Child Behavioral Checklist. DISC-IV = Diagnostic Interview Schedule for Children fourth edition. ODD = Opposition Defiant Disorder, CD = Conduct Disorder. ADHD Subtypes: I = Inattentive, H = Hyperactive, C = Combined. Values are mean (SD).

The genotype distribution conformed to a Hardy-Weinberg equilibrium (χ^2 ^= 0.42; df = 2, p = 0.81). 156 parents participated in the study and gave blood samples. Among these parents, 76 were heterozygous (M = 43 and F = 33) and transmitted the *Val *allele to their affected children in 28 occurrences, whereas this same allele was not transmitted in 29 occurrences [χ^2 ^= 0.02; df = 1, p > 0.05 (transmission disequilibrium)]. Conversely, parents transmitted the *Met *allele to their affected children in 29 occurrences, whereas this same allele was not transmitted in 28 occurrences [χ^2 ^= 0.02; df = 1, p > 0.05 (transmission disequilibrium)]. In addition, results from the QTDT revealed no evidence of linkage or within-family association between the three quantitative phenotypes and the *COMT *gene.

A one-way ANOVA performed on these data revealed no significant difference between the LL, HL, and HH genotypes according to WCST standardized perseverative error scores [F_2,97 _= 0.66, p > 0.05](Table 2) and TOL standardized total item scores [F_2,99 _= 0.97, p > 0.05](Table 2). A repeated-measure, mixed design ANCOVA performed on these data revealed no effect of genotype on SOPT raw error scores [F_2,108 _= 0.62, p > 0.05] (Table 2), TOL raw item scores [F_2,107 _= 0.35, p > 0.05], and TOL time to complete each trial [F_2,108 _= 0.04, p > 0.05]. No genotype by task interaction was observed for SOPT raw error scores [F_6,327 _= 0.39, p > 0.05], TOL raw item scores [F_11,1199 _= 1.63, p > 0.05], and TOL time to complete each trial [F_11,1210 _= 1.65, p > 0.05]. A one-way ANCOVA performed on these data revealed no effect of genotype on WCST number of categories completed [F_2,96 _= 1.94, p > 0.05], WCST number of trials to first category [F_2,96 _= 1.04, p > 0.05] and TOL number of problems solved [F_2,112 _= 1.04, p > 0.05]. No genotype effects were observed when the HL and HH genotype groups were combined into one category and contrasted with the LL genotype (recessive model) on WCST standardized perseverative error scores [F_1,98 _= 1.11, p > 0.05], WCST number of categories completed [F_1,97 _= 0.01, p > 0.05], WCST number of trials to first category [F_1,97 _= 0.36, p > 0.05], TOL standardized total item scores [F_1,100 _= 0.42, p > 0.05], TOL raw item scores [F_1,108 _= 0.22, p > 0.05], TOL time to complete each trial [F_1,109 _= 0.07, p > 0.05], TOL number of problems solved [F_1,113_= 1.33, p > 0.05] and SOPT raw error scores [F_1,109 _= 0.85, p > 0.05].

**Table 2:** Neuropsychological task performance in children with ADHD

	LL (23)	HL (66)	HH (29)	ES	p-value
WCST	96.3 (15.1)	99.1 (11.8)	100.6 (12.2)	0.31	F_2,97 _= 0.67, p = 0.52
TOL	103.3 (16.5)	99.5 (15.1)	103.8 (12.6)	0.03	F_2,99 _= 0.97, p = 0.38
SOPT	13.5 (6.9)	15.1 (8.8)	15.8 (8.2)	0.31	F_2,108 _= 0.62, p = 0.54

WCST = Wisconsin Card Sorting Test standardized perseverative error score (LL: n = 21, HL: n = 56, HH: n = 23). TOL = Tower of London standardized score (LL: n = 20, HL: n = 55, HH: n = 27). SOPT = Self Ordered Pointing Task error score (LL: n = 23, HL: n = 63, HH: n = 26). ES = Effect size for LL vs. HH. Values are mean (SD).

## Discussion

Previous studies have identified an association between the *COMT *polymorphism and a variety of indices reflecting executive control both in healthy [36,37] and schizophrenic adults [39-41]. The *COMT *appears to be important to the regulation of dopamine metabolism within the PFC [33-35]. Since the PFC and dopamine pathways have been hypothesized to play an important role in the pathogenesis of ADHD [9-11,60,61]), we conducted this study in an attempt to test whether the *COMT Val*^108/158^*Met *polymorphism, which is known to be associated with a significant change in the catabolic capacity of this enzyme, modulates the risk for ADHD or various indices of executive control. Contrary to our expectations and findings in both healthy [36,37] and schizophrenic adults [39-41], an association between the *Val*^108/158 ^*Met *functional polymorphism of the *COMT *gene and neuropsychological task performance reflecting executive control was not observed in children with ADHD. This result is consistent with the findings of a recent case-control study conducted by Mills et al. (2004), which, to our knowledge, is the only other study to investigate the relationship between the *COMT Val*^108/158^*Met *polymorphism and neuropsychological task performance in children with ADHD [62]. However, this study did not include the WCST, the measure responsible for producing the most consistent results in the previous literature. In addition, we did not identify a biased transmission of either of the two alleles from parents to affected offspring.

The absence of an association between the *COMT Val*^108/158^*Met *polymorphism and behavioral indices of executive function in children with ADHD may be explained by the young age of the population of patients included in the present study. Indeed it is possible that, due to age-related changes in the functional importance of the *COMT *within the prefrontal cortex, this association is observable only in adults. This possibility is supported by data in both rats [63-65] and humans [66,67] suggesting that monoamine content and metabolism decrease with age. This age-related decrease may render functions dependent on monoamine content more prone to be dysfunctional at an older age. In addition, evidence from rat studies has indicated a positive correlation between aging and *COMT *activity [68-70]. This observation may suggest that the implication of the *COMT *in the catabolism of dopamine is developmentally regulated, with children relying less on this catabolic pathway than adults. Conversely, it has been reported that DAT density is inversely correlated with age [71]. Taken together, the presence of an inverse and direct correlation between age and DAT density on the one hand and *COMT *activity on the other hand, may suggest that dopamine metabolism relies more on the DAT than on *COMT *activity in children compared to adults. This hypothesis is compatible with the fact that several studies have identified an association between the DAT [9,60,72-74], but not the *COMT*, gene and ADHD.

It is also possible that the negative result observed in the present study is due to a type II error (false negative) secondary to the lack of power of our sample to detect an association. However, using results from the WCST, the variable for which relevant genetic data already exists, we conducted a power analysis and determined that our sample size has sufficient power (80% at α = .05) to detect a mean difference of 11.2 on this measure. Furthermore, it is possible that some of the tests used in our assessment are mediated by the PFC but insensitive to PFC DA levels [75].

An additional limitation of the present study is that some genotype groups included few subjects. Increasing the sample size to achieve larger genotype groups would be necessary to reach firmer conclusions. This is particularly true for female subjects who were significantly underrepresented in the study (as is common to most clinical studies of ADHD). In order to generalize these negative results to females, a more comparable gender representation is required, particularly in view of some previous research indicating that the allelic distribution of the COMT may be gender dependent [59].

## Conclusions

This study does not support the involvement of the *Val*^108/158 ^*Met *polymorphism of the *COMT *gene in increasing the risk for ADHD or in modulating several indices of executive functions in children with ADHD. This result is contrary to previous findings in both healthy and schizophrenic adults and may be related to developmental specificities.

## Competing interests

The author(s) declare that they have no competing interests.

## Authors' contributions

ET performed the data analysis and drafted the manuscript. NG was involved in the conception of the study and provided clinical support. LBA provided clinical support and aided in data collection. PL provided clinical support. VM aided in neuropsychological testing and data collection. RD and ATZ performed the genotyping for the study and aided in data management. MTS coordinated the clinical aspects of the study and was involved in data management. CB provided clinical support. RJ was responsible for the conception of the study, drafting of the manuscript, and supervision of the research project.

## Pre-publication history

The pre-publication history for this paper can be accessed here:


